# Supplementary material for: Dynamic network inference and association computation discover gene modules regulating virulence, mycotoxin and sexual reproduction in Fusarium graminearum
Source: BMC Genomics. 2020 Feb 24;21:179. doi: 10.1186/s12864-020-6596-y (PMC7041293; doi:10.1186/s12864-020-6596-y)
Supplement: Supplementary file 5 — Additional file 5: Figure S1. System framework and main steps in constructing a modularized gene regulatory network of Fusarium graminearum. [file 12864_2020_6596_MOESM5_ESM.docx]

**
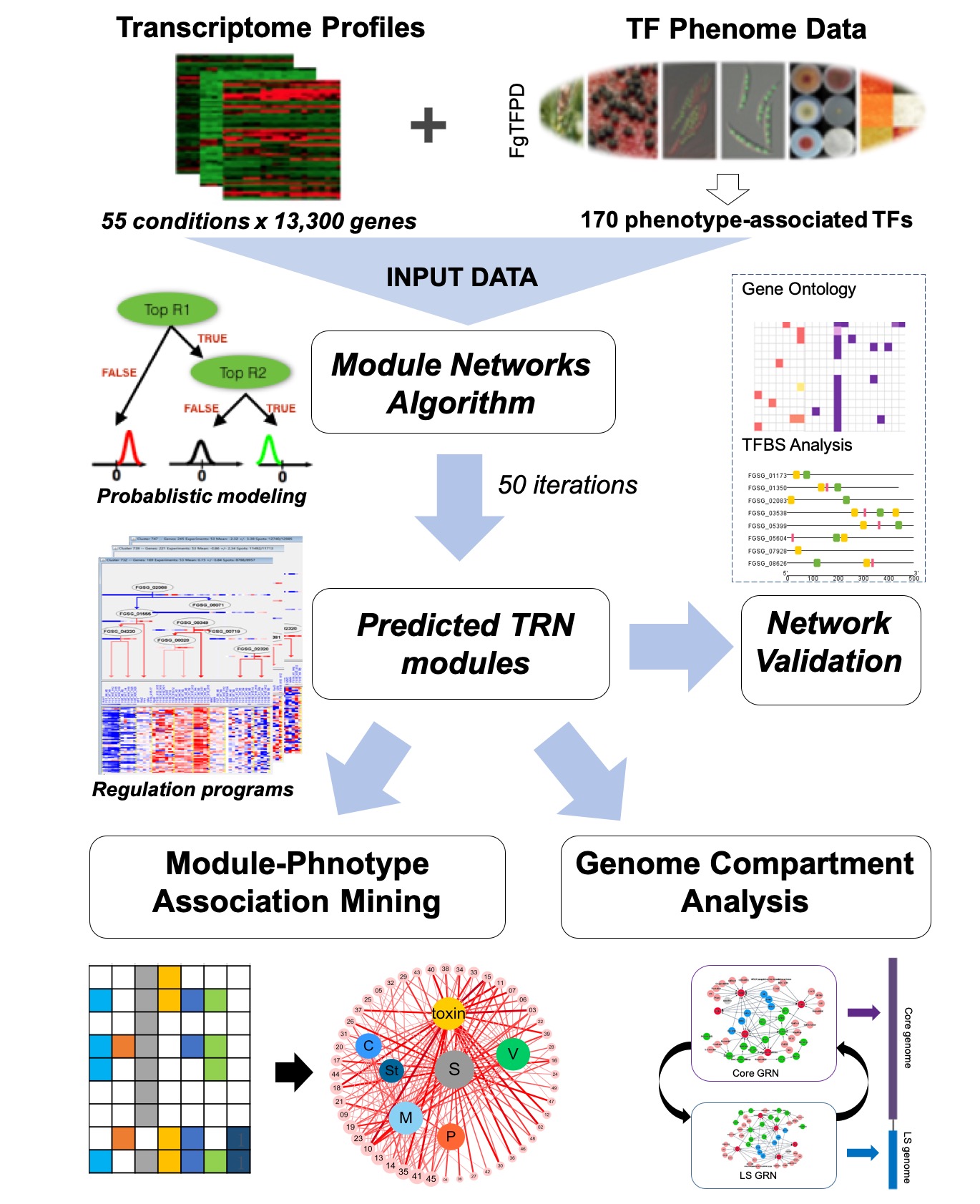
**

**Figure S1. System framework and main steps in constructing a modularized gene regulatory network of *Fusarium graminearum***. Combined input consisting of gene expression data and information on candidate regulators was provided to the module networks algorithm (Segal *et al.* 2003). The inferred modules were then subjected to a robust validation process involving functional annotations and transcription factor binding site analysis. Then, the modules were analyzed for their phenotypic associations using an in-house computational method. Finally, the genome compartmentalization of the regulatory network was analyzed to provide evolutionary insights into the gene circuits.
