# Supplementary material for: Dynamic network inference and association computation discover gene modules regulating virulence, mycotoxin and sexual reproduction in Fusarium graminearum
Source: BMC Genomics. 2020 Feb 24;21:179. doi: 10.1186/s12864-020-6596-y (PMC7041293; doi:10.1186/s12864-020-6596-y)
Supplement: Supplementary file 8 — Additional file 8: Figure S2. Regulatory programs for all 49 regulatory modules in F. graminearum. [file 12864_2020_6596_MOESM8_ESM.docx]

Fig. S2-1 to Fig. S2-49 (Figures for all 49 regulatory modules: M01-M49)


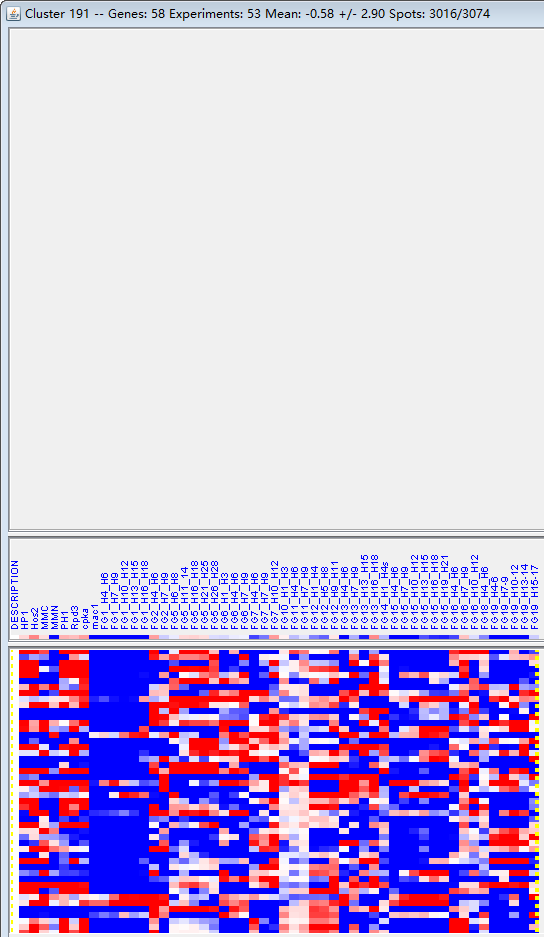

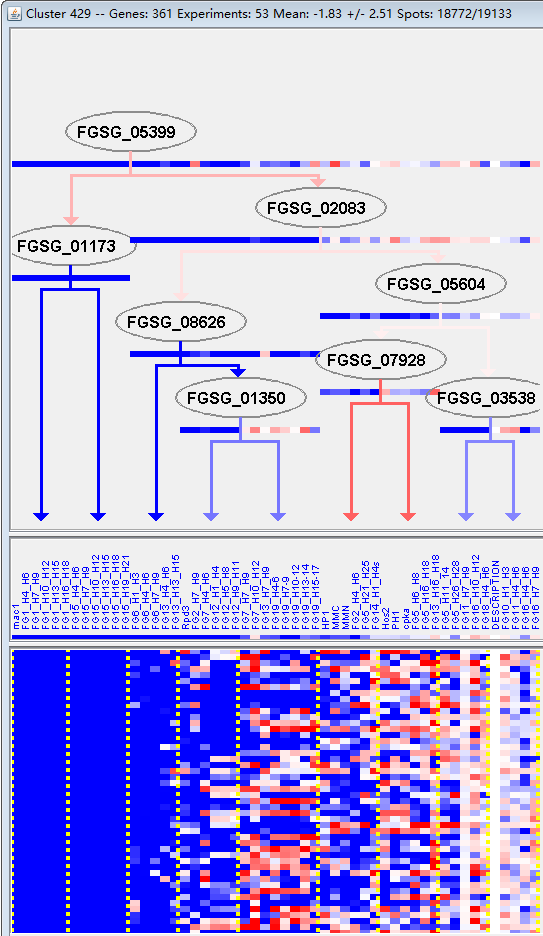

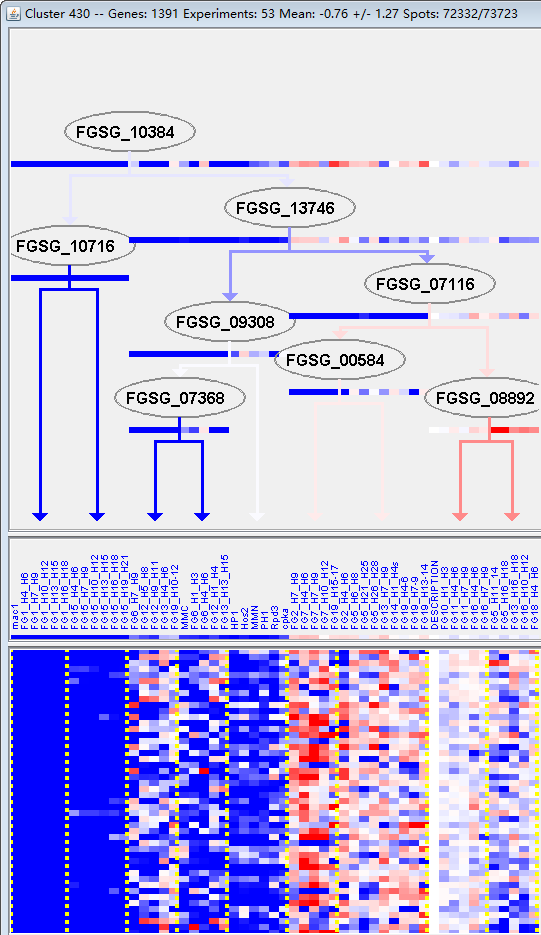

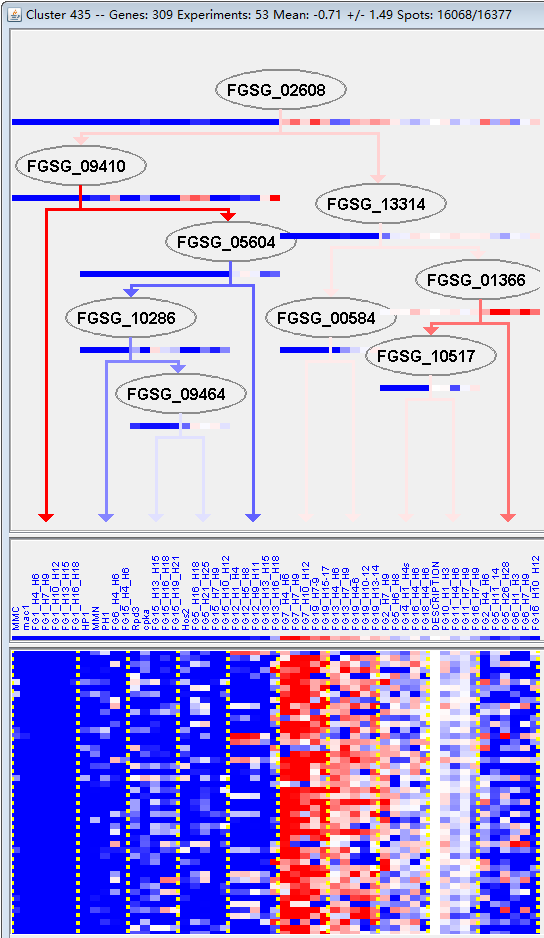


**M02**

**M01**

**M04**

**M03**


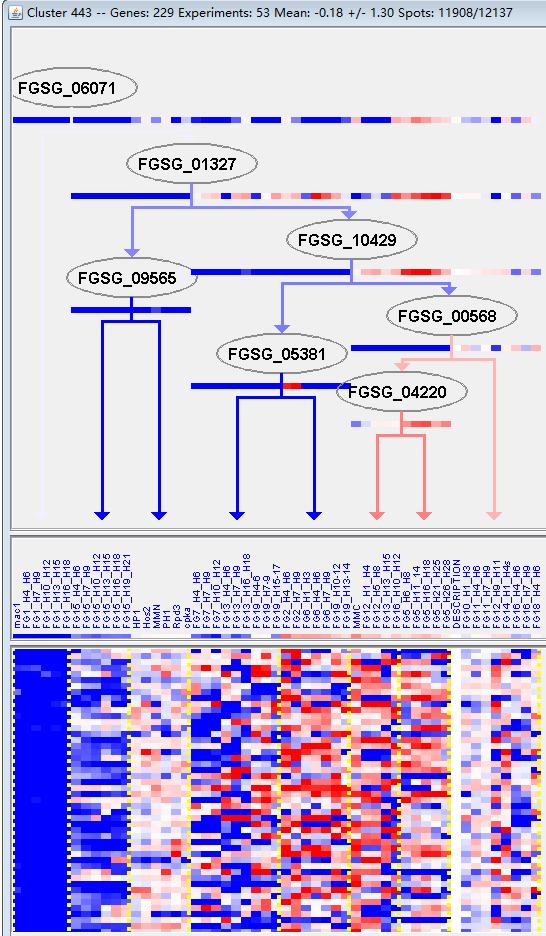

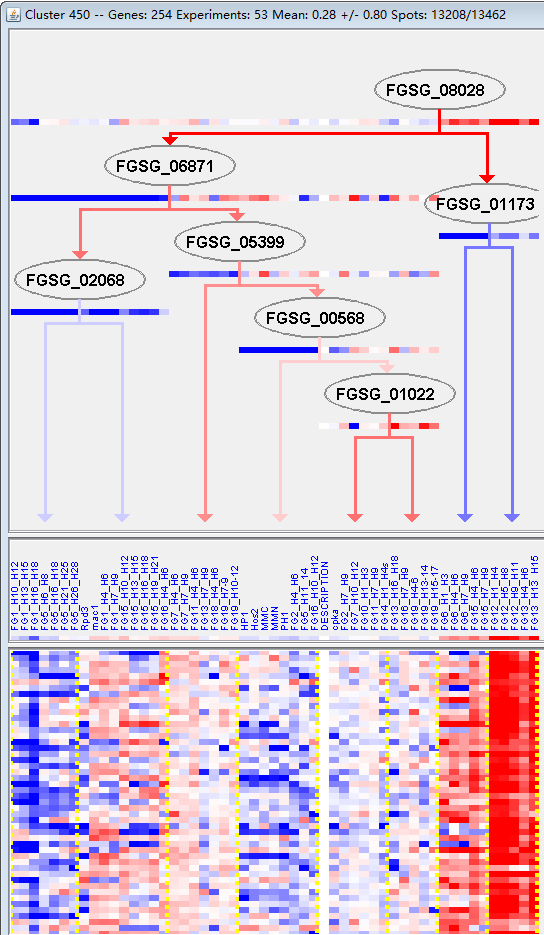


**M05**

**M06**


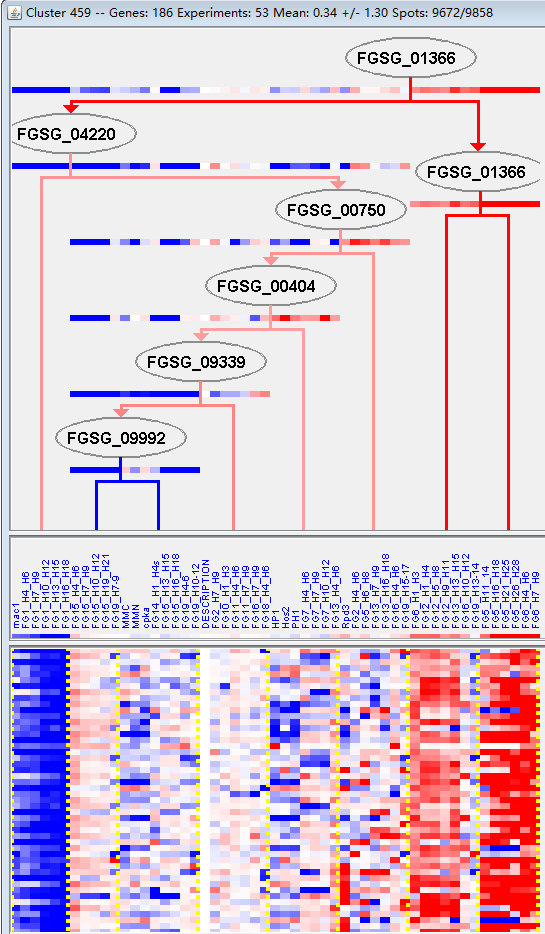

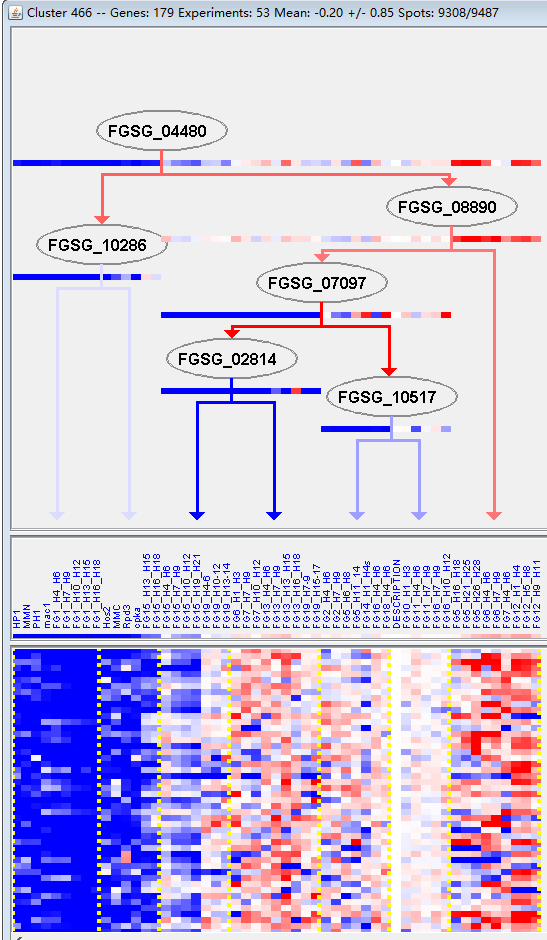


**M07**

**M08**


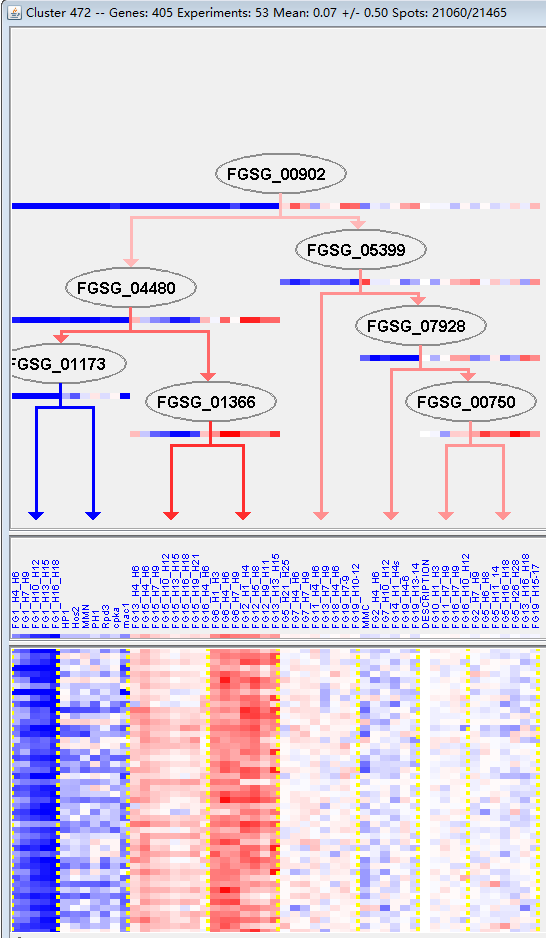

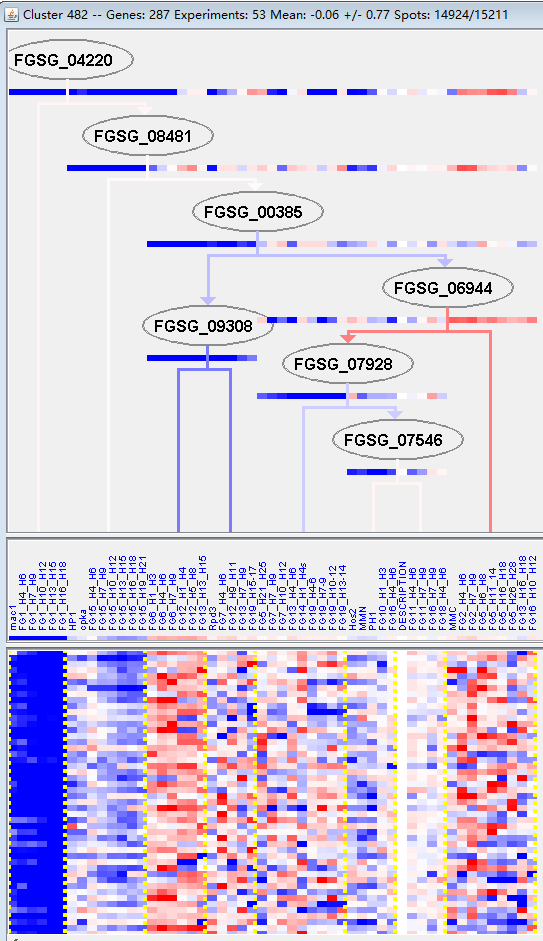


**M9**

**M10**


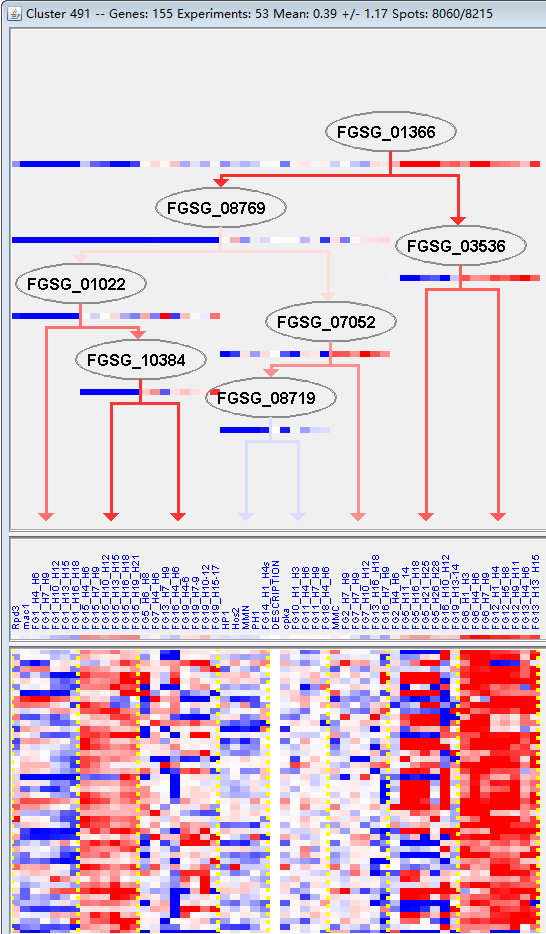

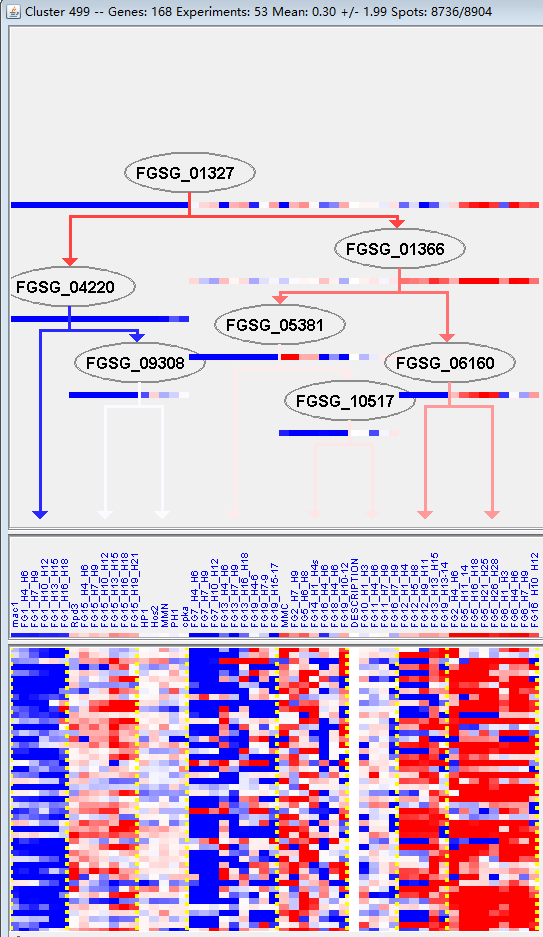


**M12**

**M11**


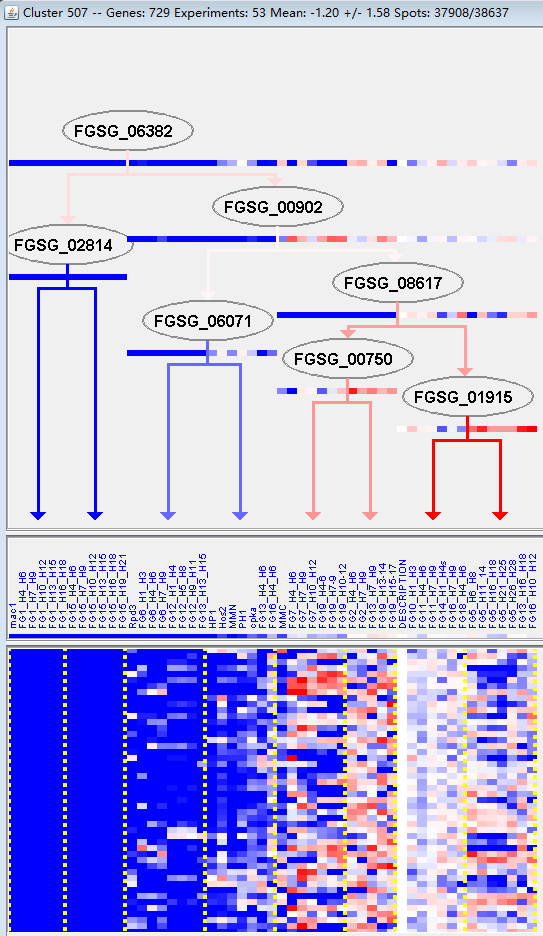

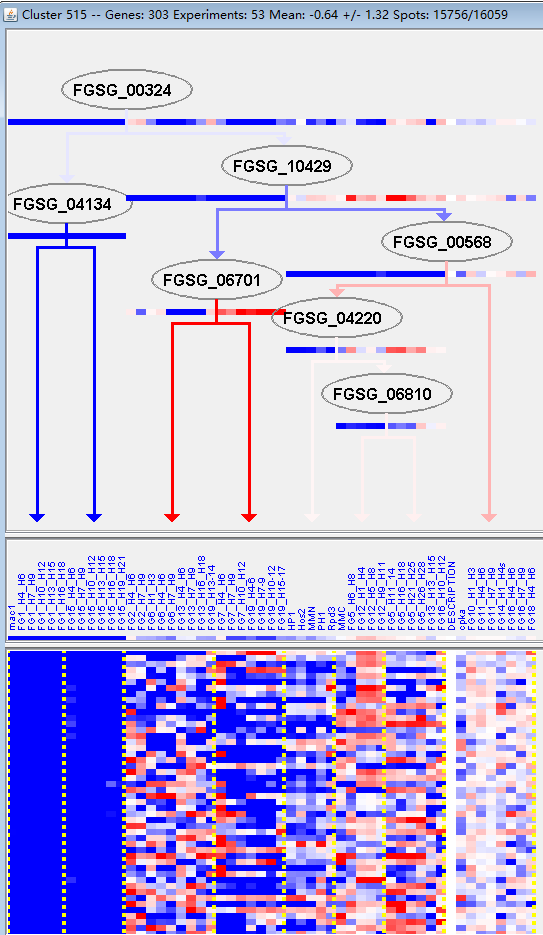


**M14**

**M13**


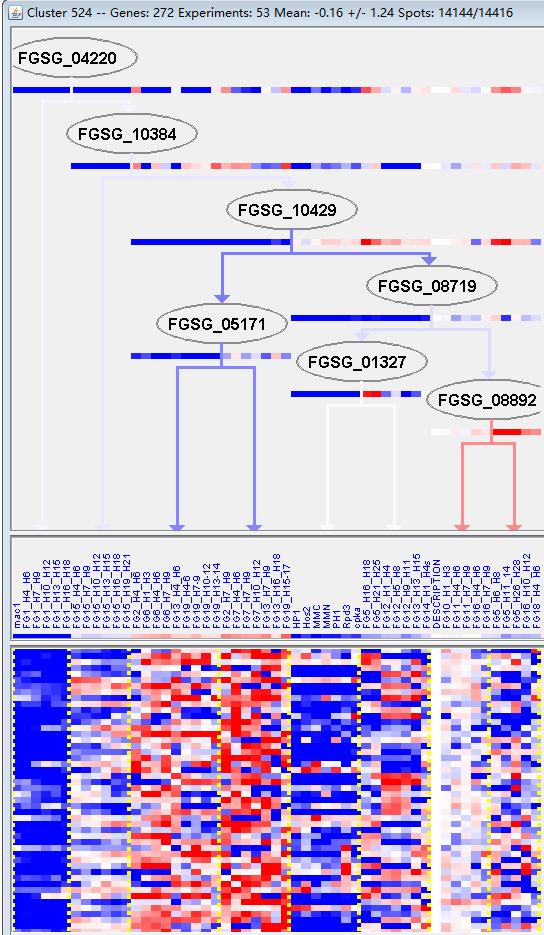

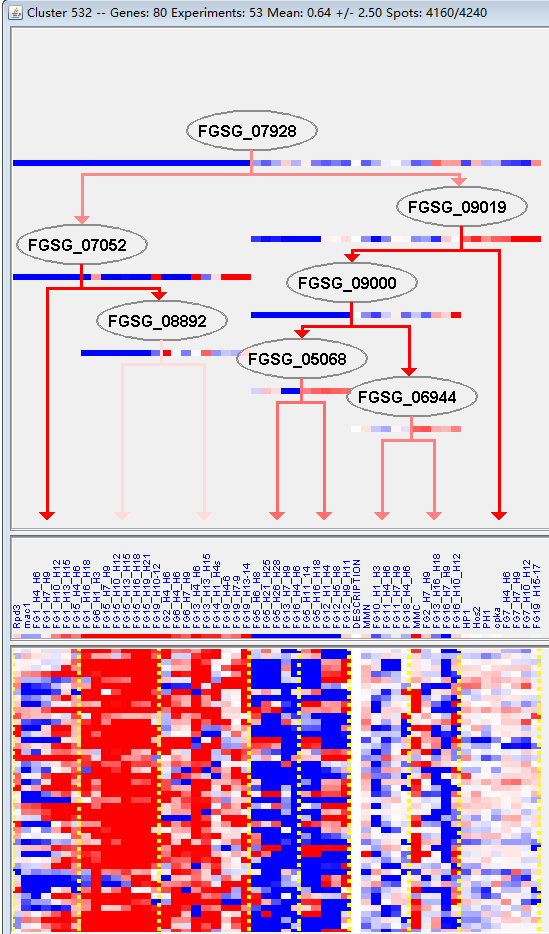


**M16**

**M15**


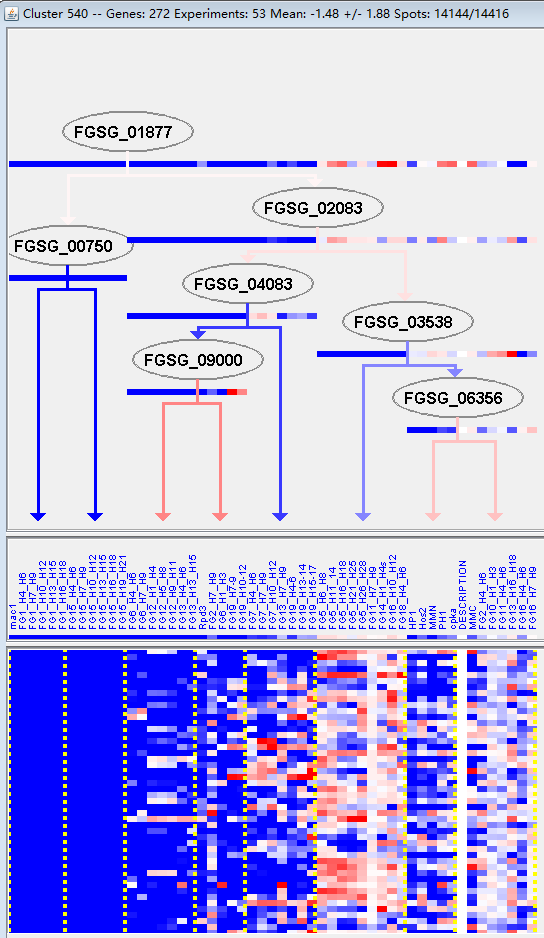

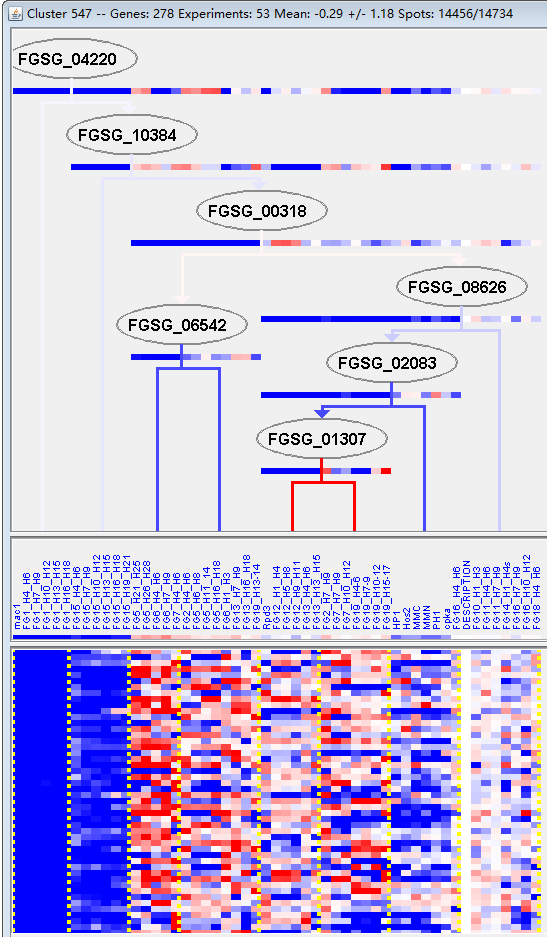


**M18**

**M17**


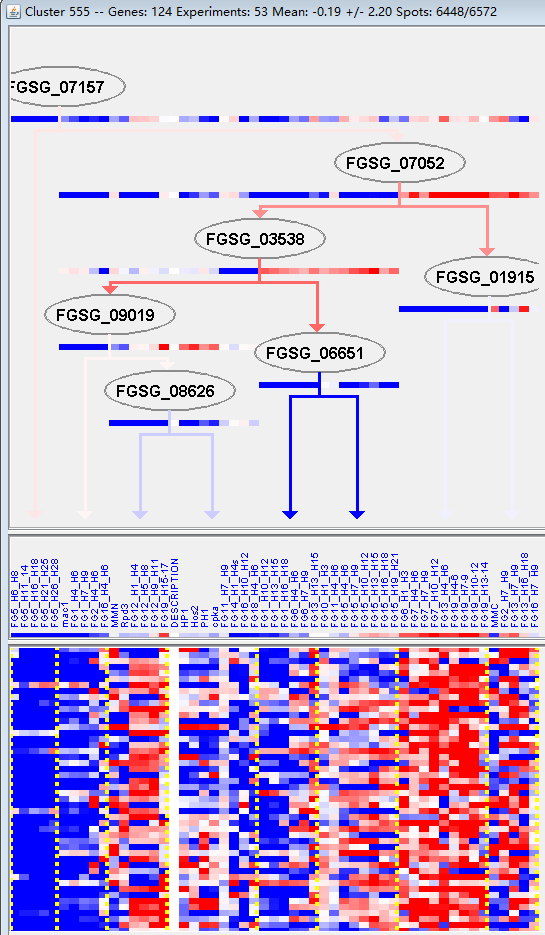

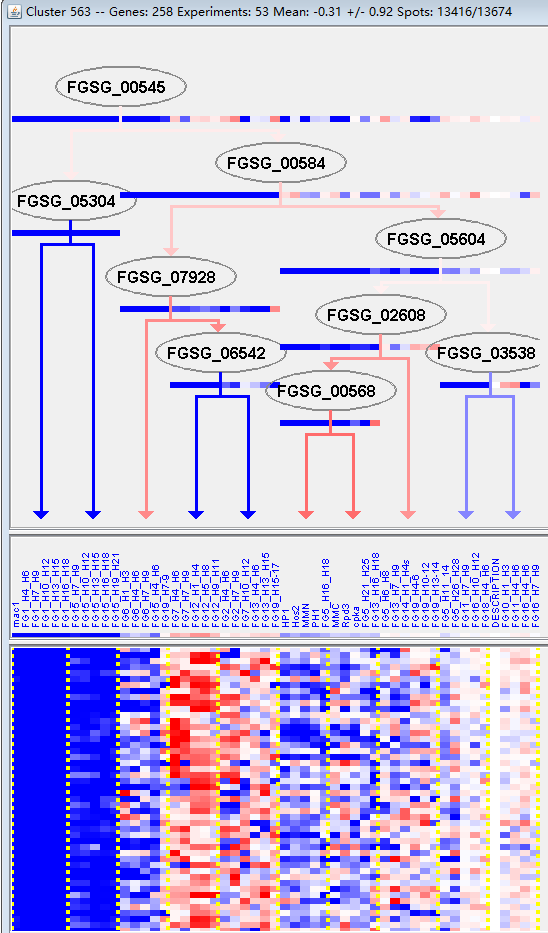


**M19**

**M20**


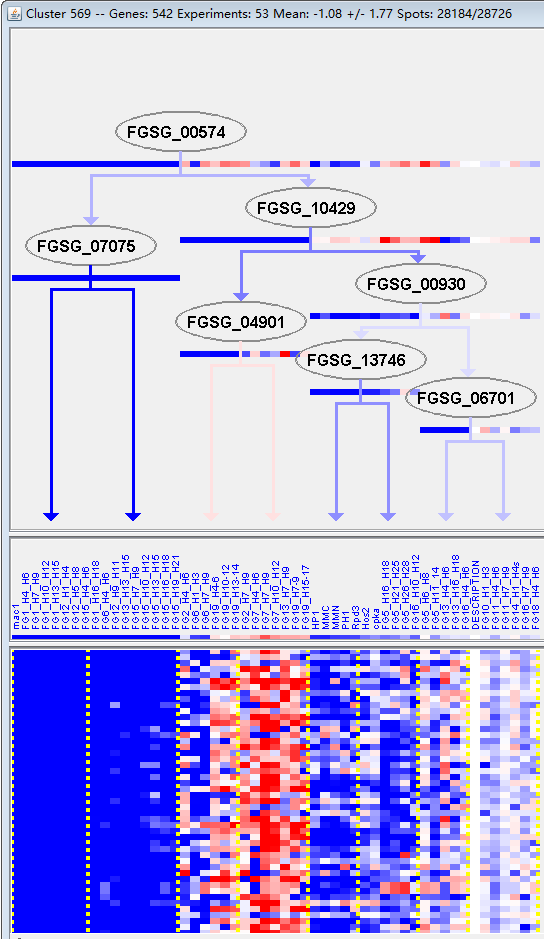

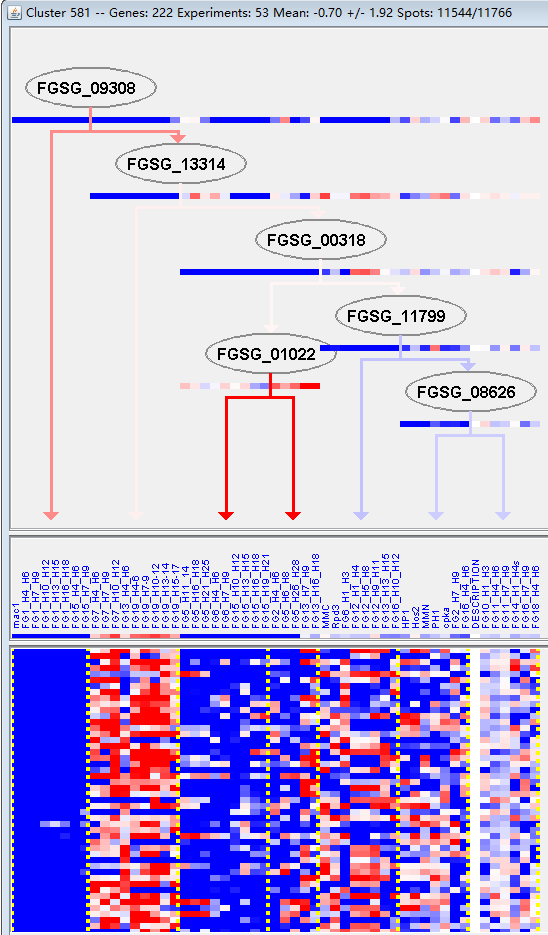


**M22**

**M21**


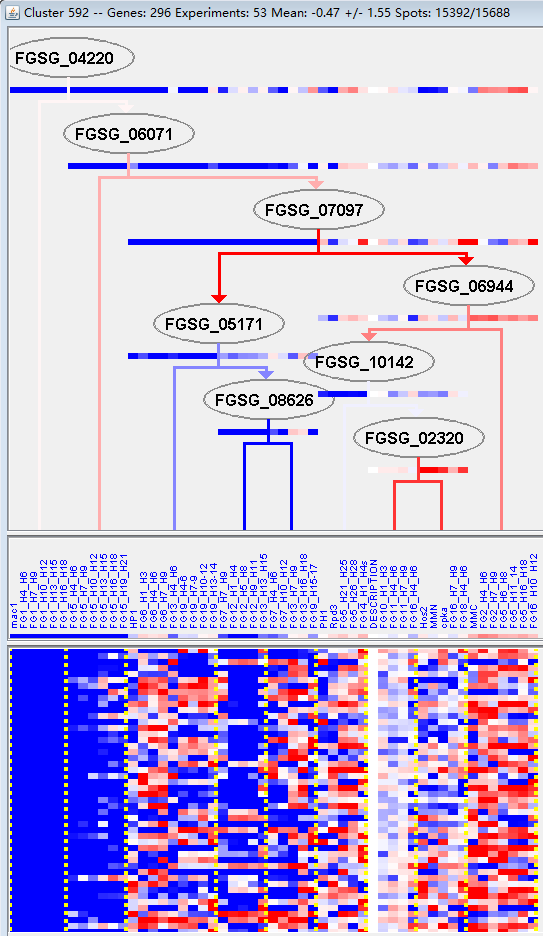

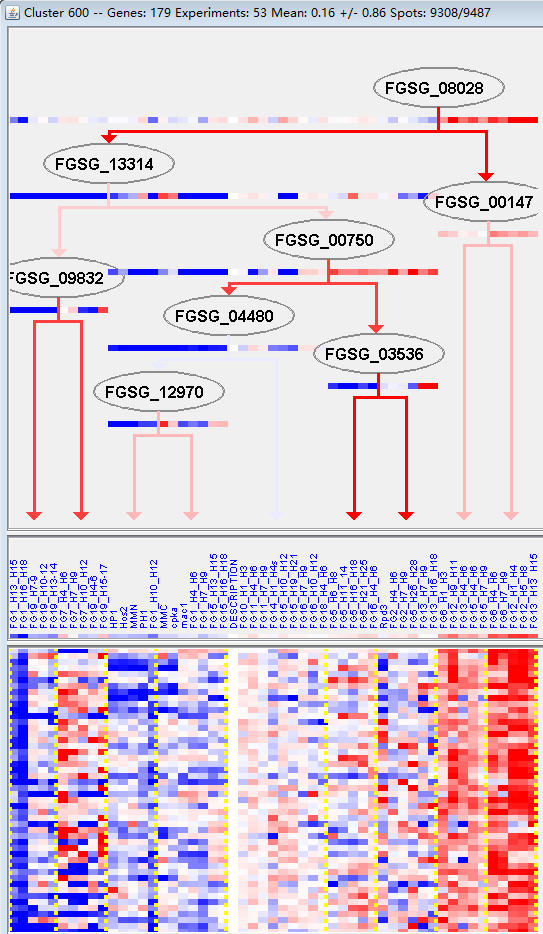


**M24**

**M23**


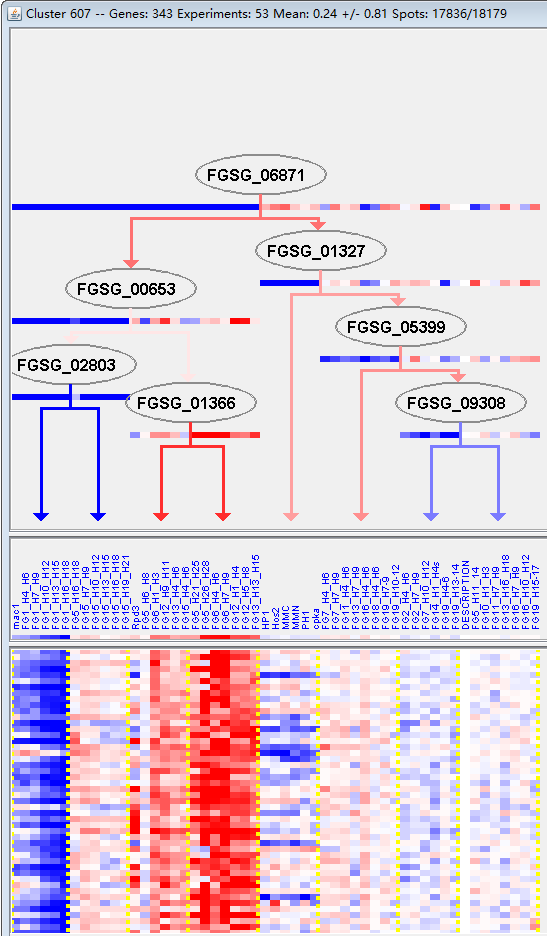

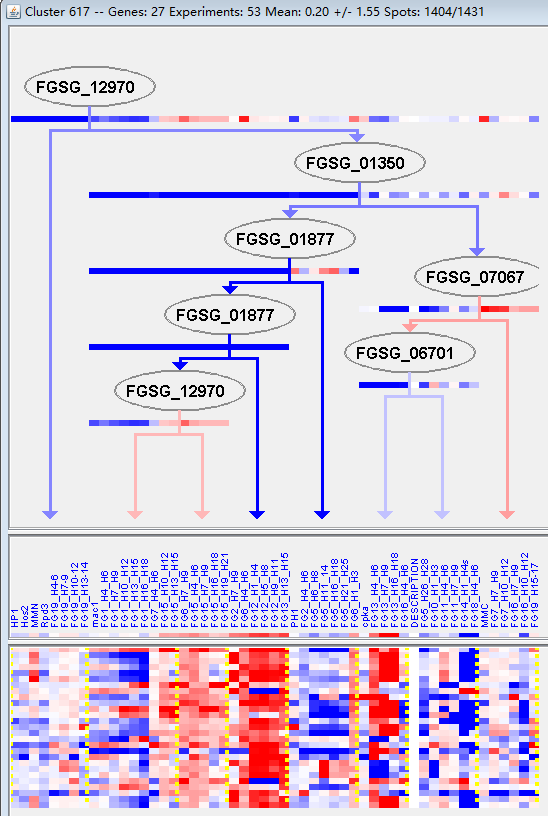


**M25**

**M26**


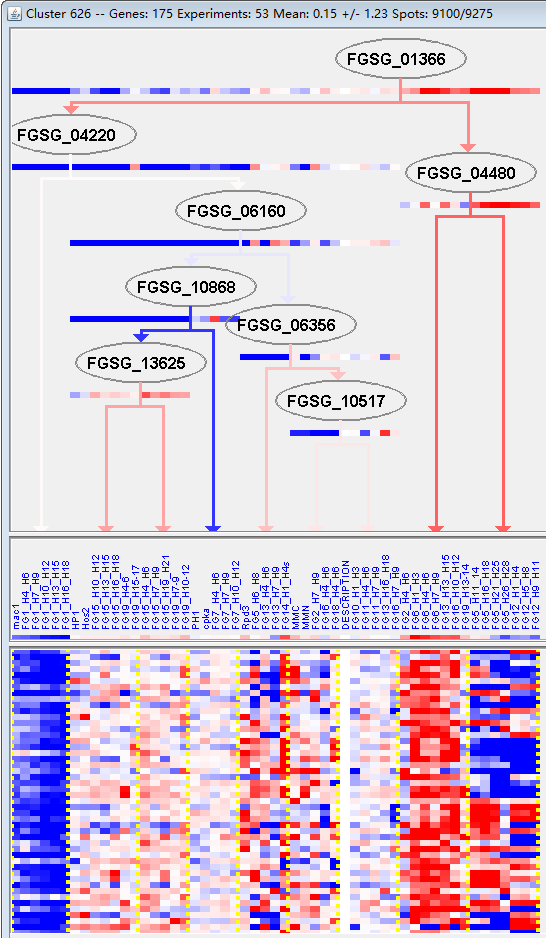

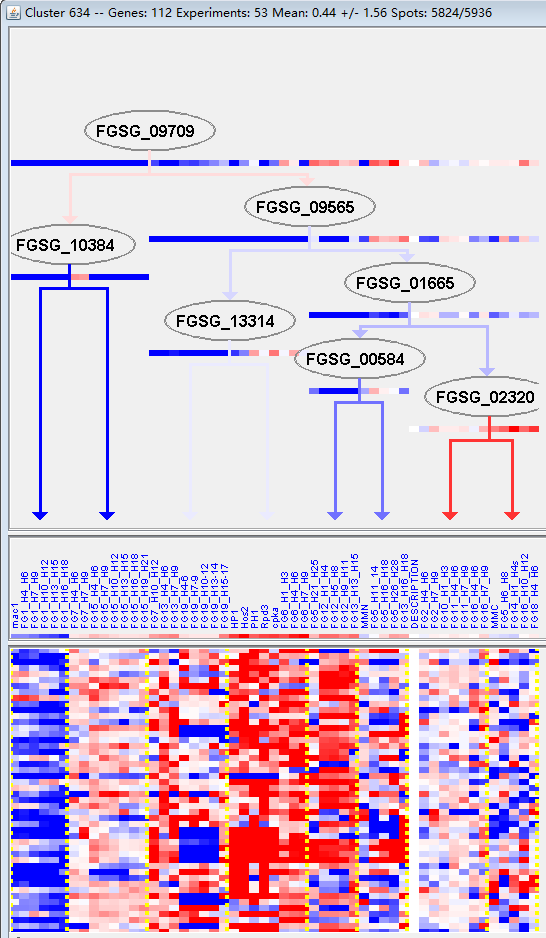


**M28**

**M27**


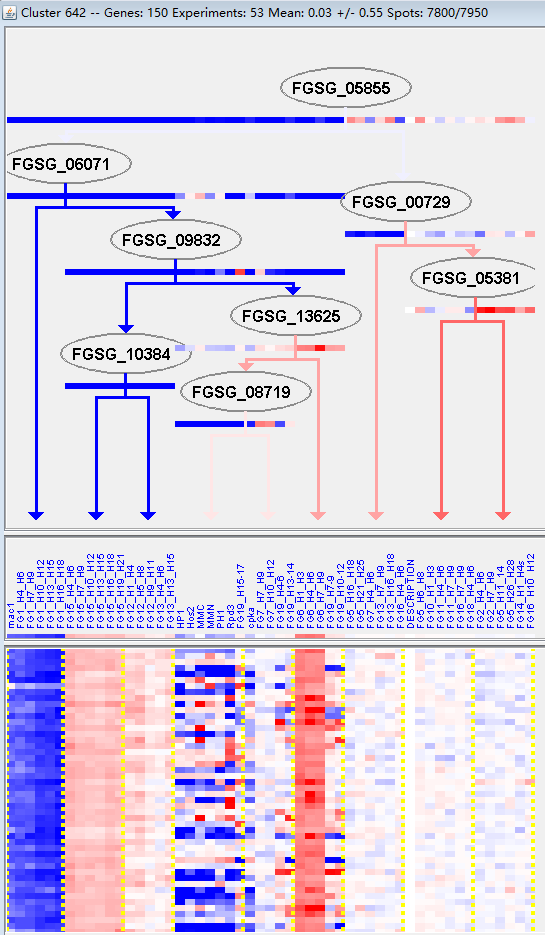

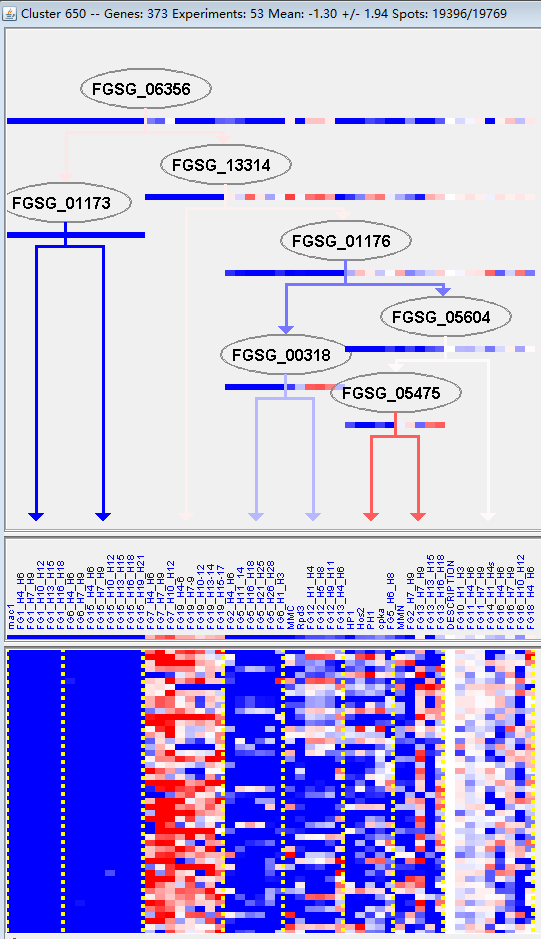


**M29**

**M30**


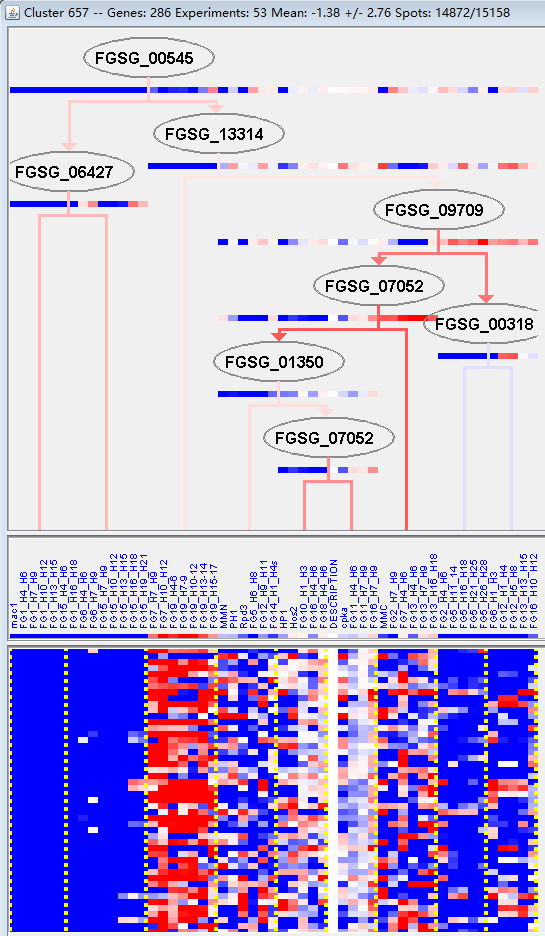

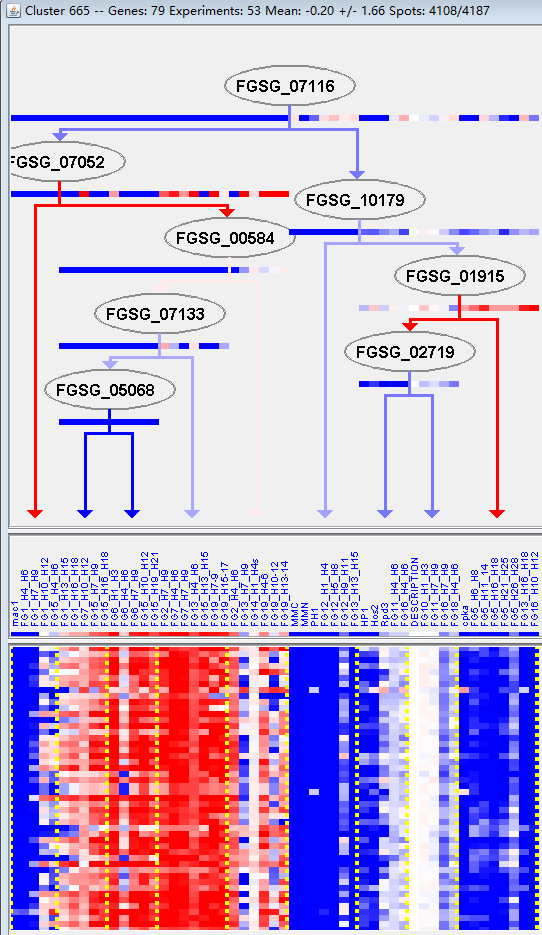


**M32**

**M31**


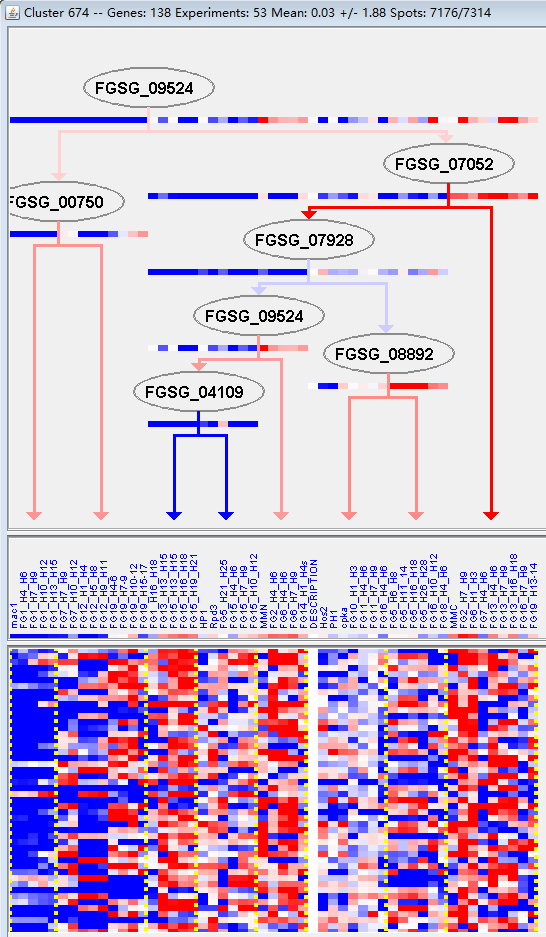

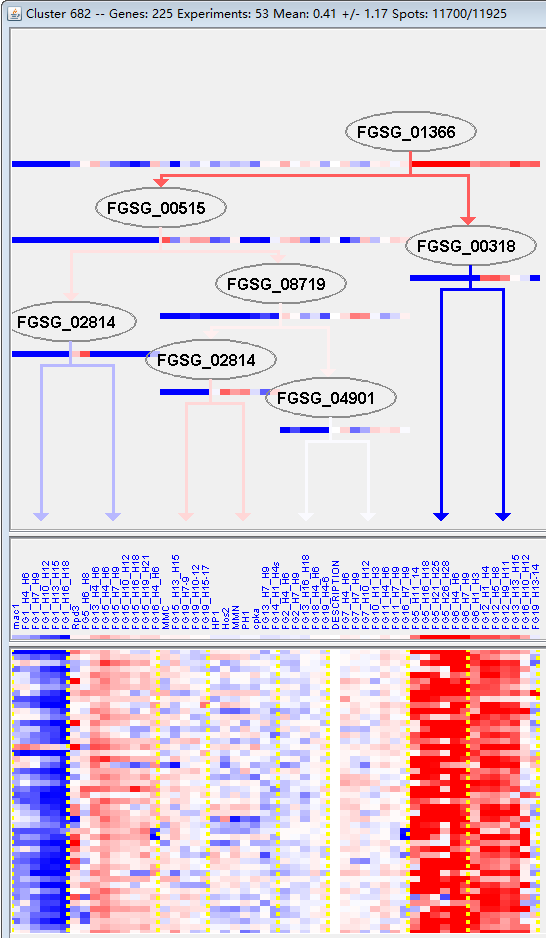


**M34**

**M33**


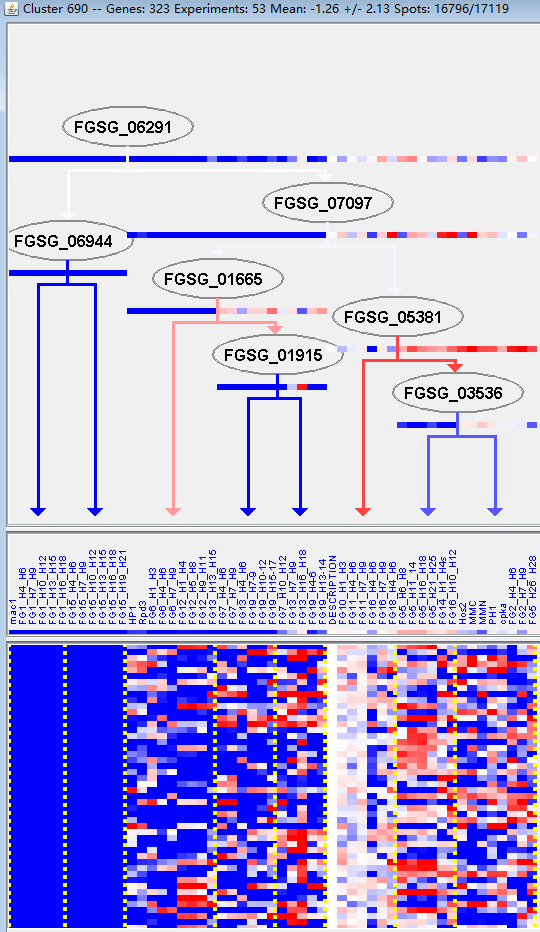

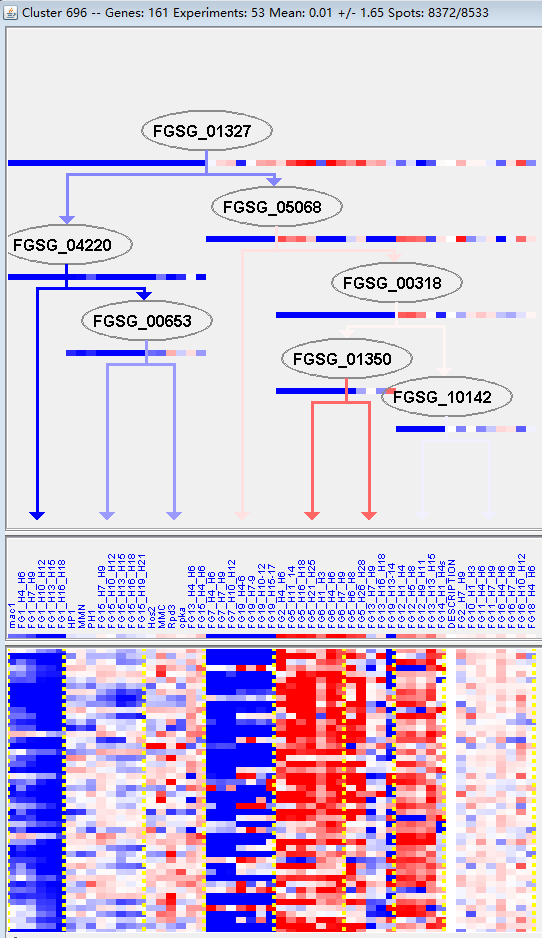


**M35**

**M36**


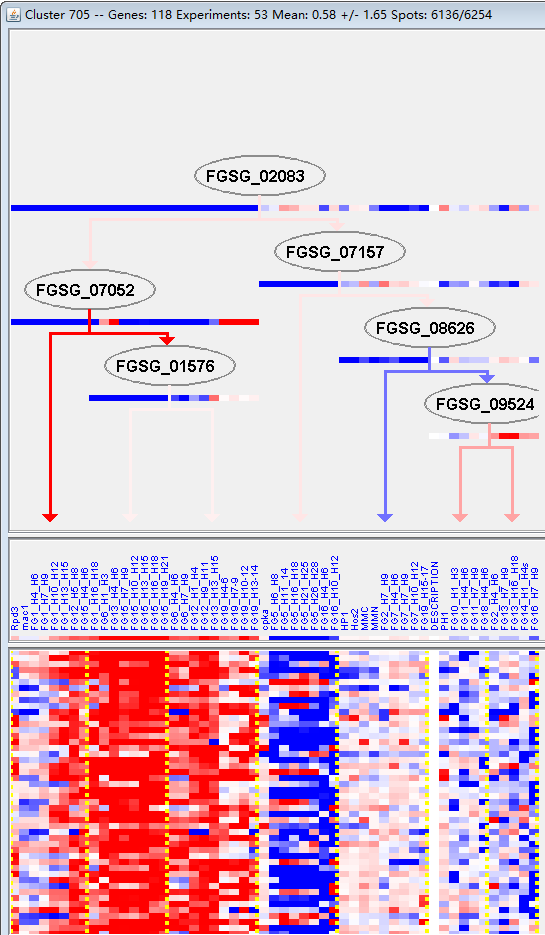

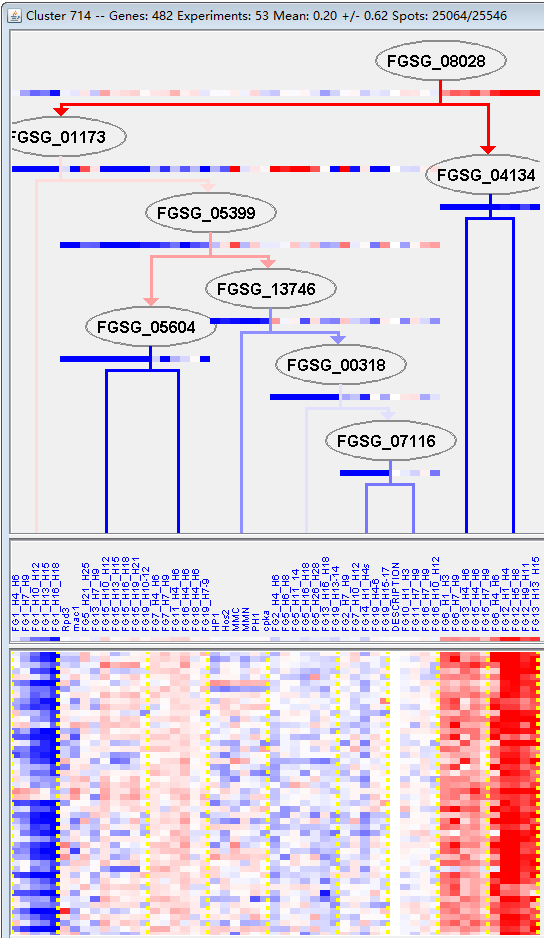


**M37**

**M38**


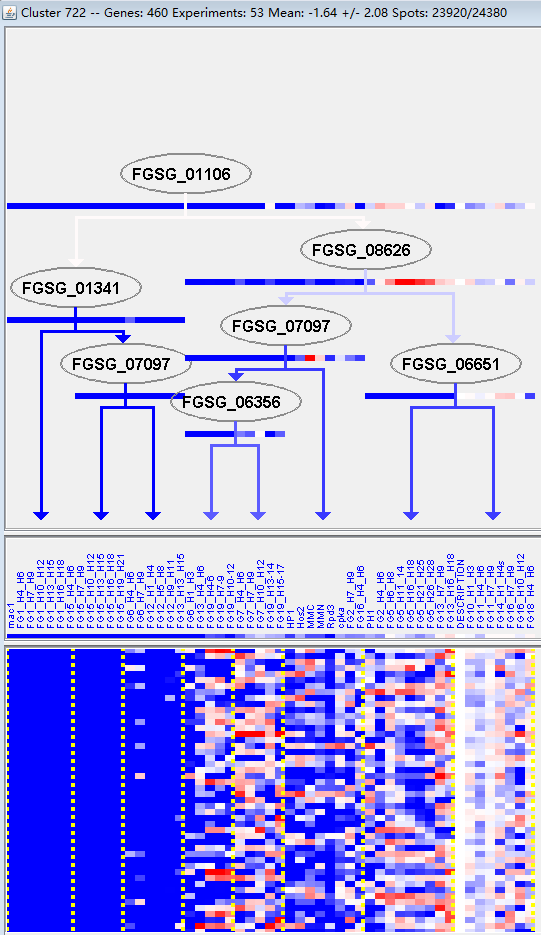

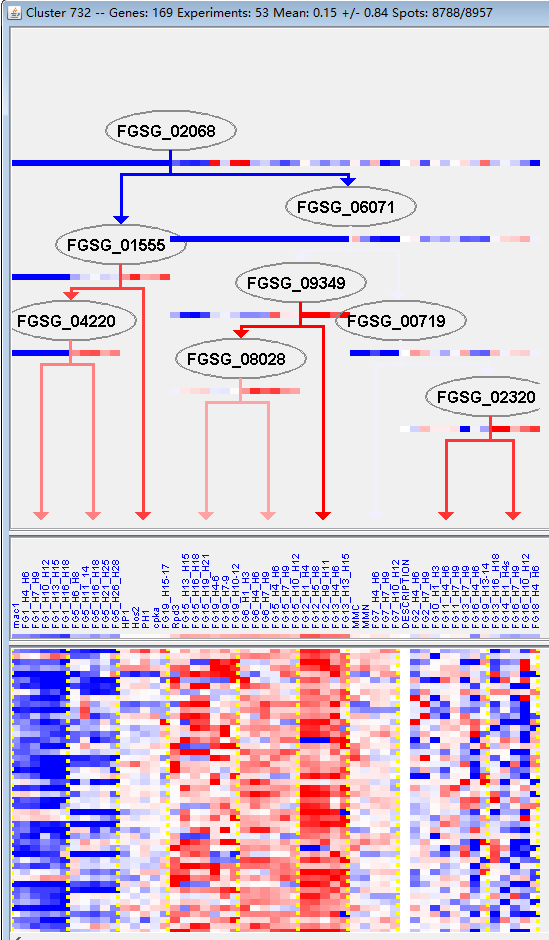


**M39**

**M40**


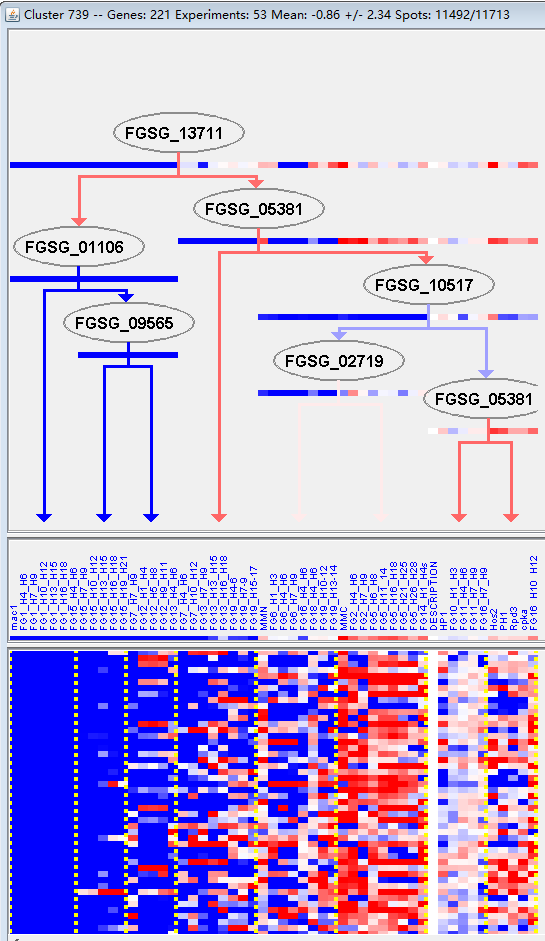

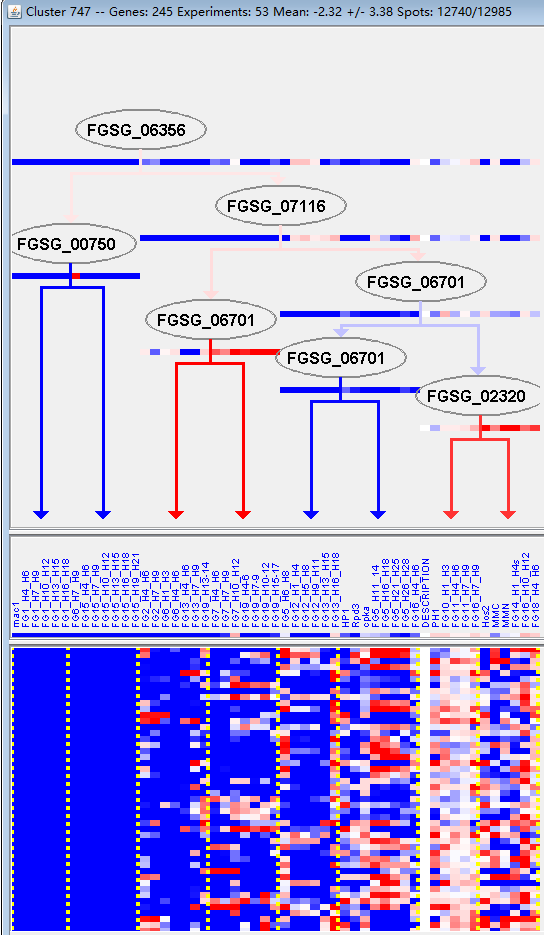


**M41**

**M42**


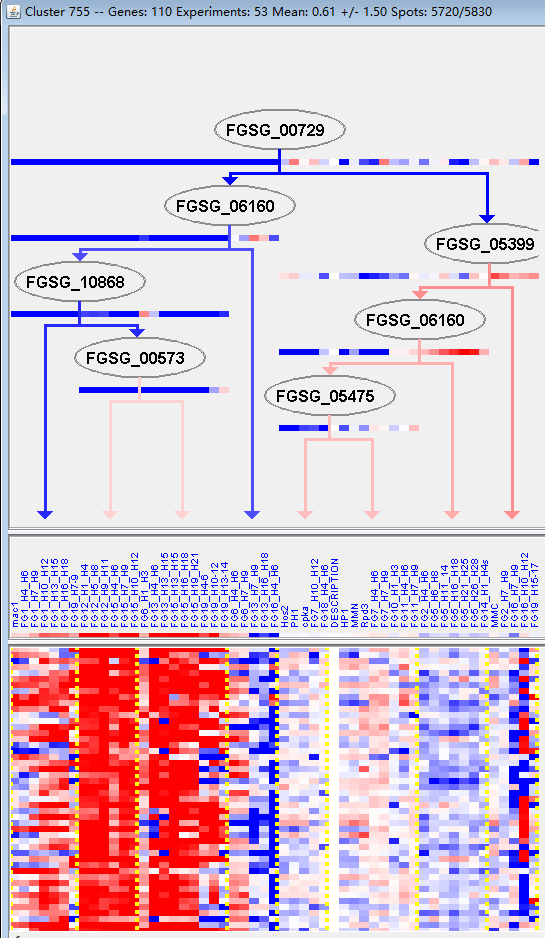

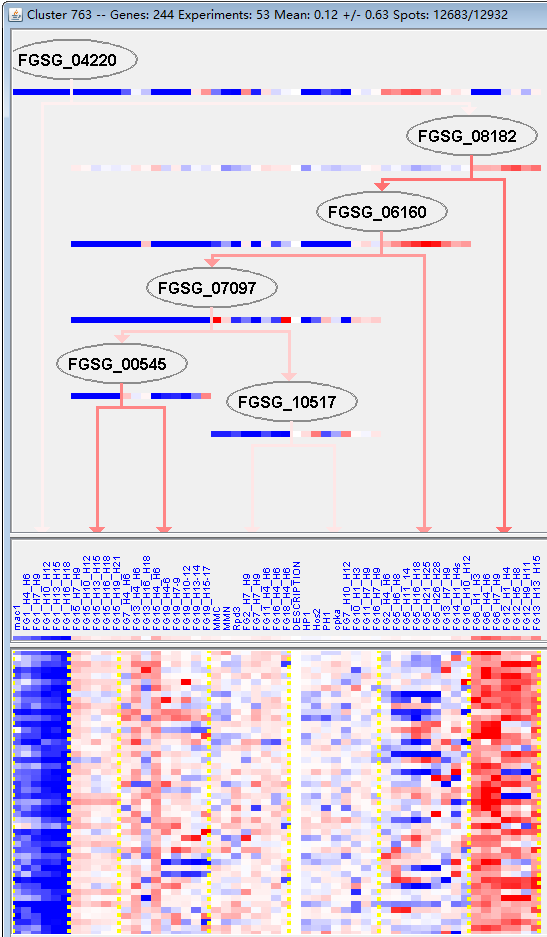


**M43**

**M44**


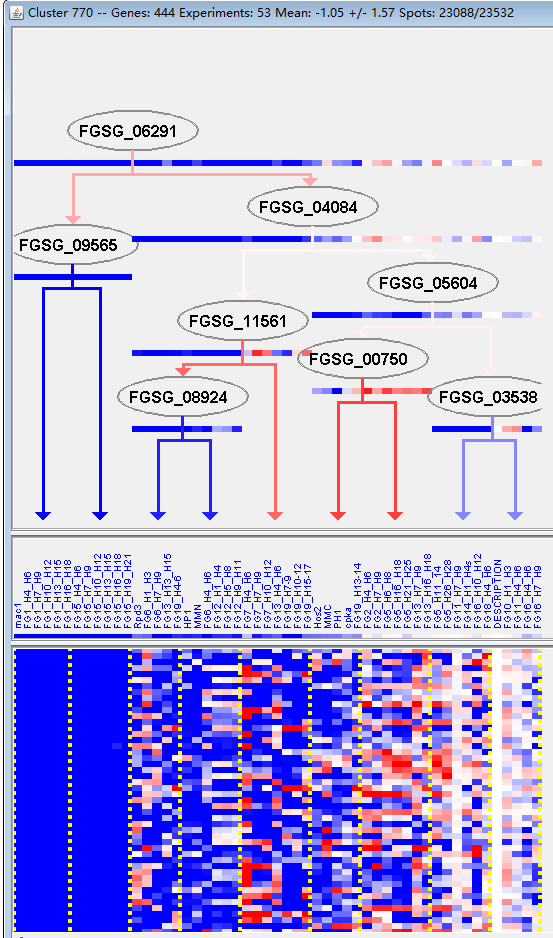

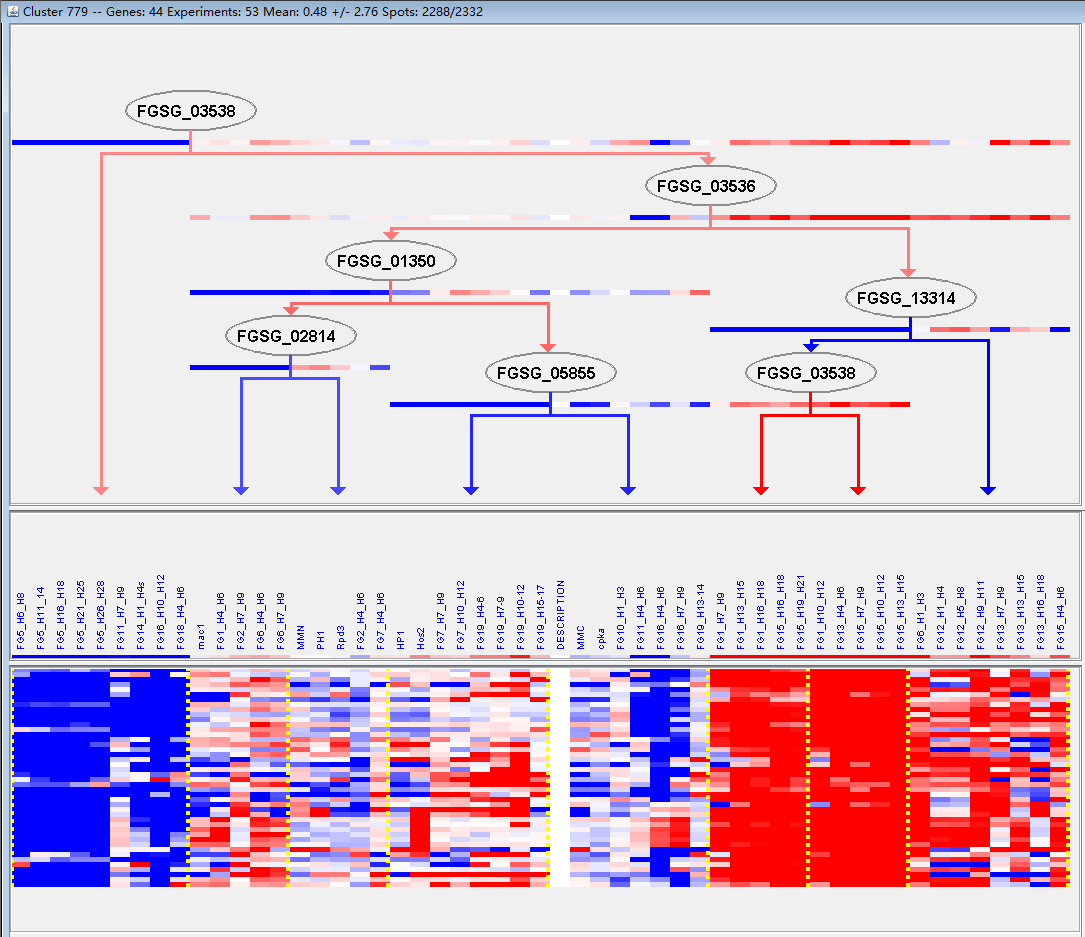


**M45**

**M46**


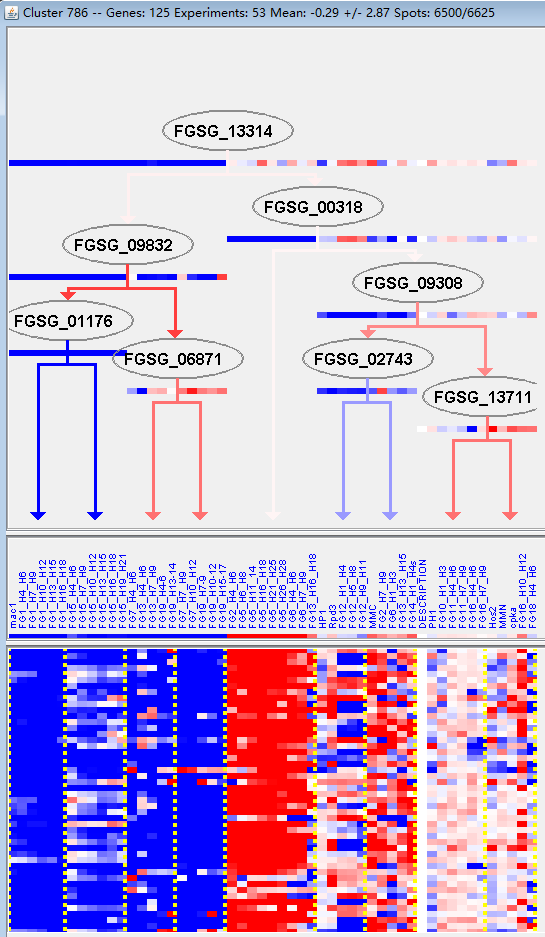

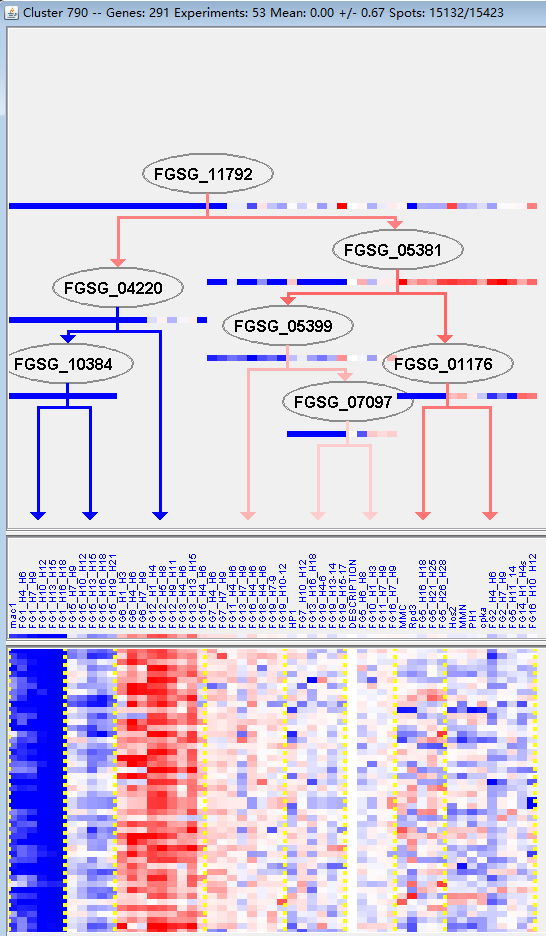


**M47**

**M48**


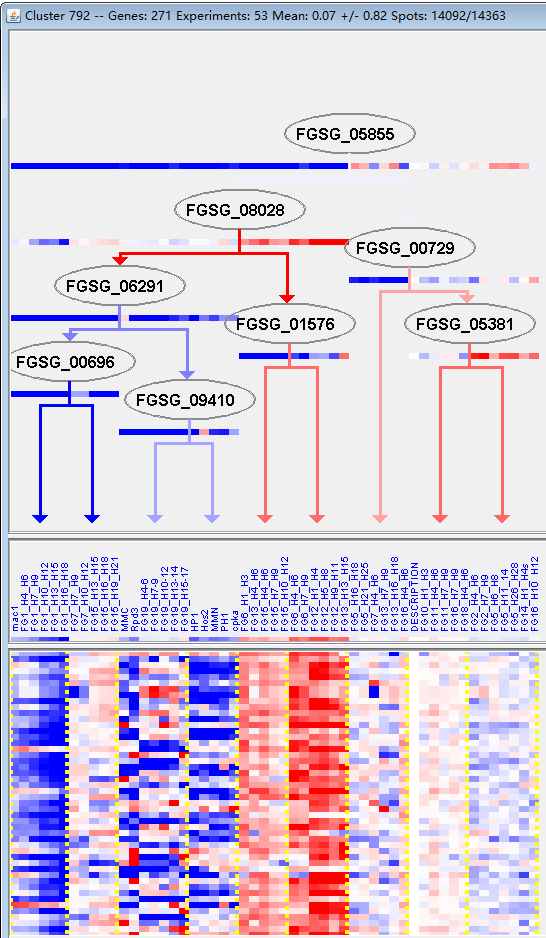


**M49**
